# Assessment of Medication Regimen Complexity and Adherence in Chronic Obstructive Pulmonary Disease Patients at a Tertiary Care Teaching Hospital

**DOI:** 10.1002/puh2.70449

**Published:** 2026-11-07

**Authors:** Kabita Bhandari, Nirmal Raj Marasine, Chakra Dev Bist, Poonam Pant

**Affiliations:** ^1^ Department of Pharmacy CiST College Pokhara University Kathmandu Nepal; ^2^ Pharmacist, Himmat Medical Hall, Kathmandu, Nepal Kathmandu Nepal

**Keywords:** chronic obstructive pulmonary disease, medication adherence, medication prescribed, medication regimen complexity, tertiary care hospital

## Abstract

**Background:**

Chronic obstructive pulmonary disease (COPD) is a leading cause of morbidity and mortality worldwide. Medication regimen complexity and poor adherence are major barriers to optimal management, yet limited evidence exists in Nepal. This study aimed to assess medication regimen complexity and adherence among COPD patients attending a tertiary care hospital in Nepal.

**Methods:**

A hospital‐based cross‐sectional study was conducted from November 2023 to May 2024 at a tertiary care teaching hospital in Nepal. A total of 174 COPD patients aged ≥40 years were enrolled using convenience sampling. Medication regimen complexity was assessed using the validated 65‐item Medication Regimen Complexity Index (MRCI), and adherence was evaluated with the Nepali version of the Morisky Medication Adherence Scale (MMAS‐8). Pearson's chi‐square test and binary logistic regression were performed at a 5% level of significance to examine factors associated with medication nonadherence.

**Results:**

Of the total, 46.6% demonstrated suboptimal adherence, strongly associated with smoking (adjusted odds ratios [aOR]: 7.5; 95% CI: 2.82–19.90; *p* < 0.001) and alcohol use (aOR: 3.66; 95% CI: 1.578–8.49; *p* = 0.003). COPD regimens exhibited higher complexity (mean MRCI: 10.296 ± 5.4) than non‐COPD medications (3.66 ± 3.478). High COPD MRCI (aOR: 25.8; 95% CI: 14.7–38.5; *p* = 0.03) and high patient‐level MRCI (aOR: 15.6; 95% CI: 6.7–33.9; *p* < 0.001) were significant predictors of nonadherence.

**Conclusion:**

COPD‐specific regimens were found to be more complex, and higher regimen complexity together with lifestyle factors, such as smoking and alcohol consumption, were strong determinants of nonadherence. Simplifying regimens through fixed‐dose combinations, pharmacist‐led interventions, and behavioral counseling may improve medication adherence and treatment outcomes in COPD patients.

## Graphical abstract

Among 174 patients with COPD, 46.6% demonstrated suboptimal medication adherence. Greater medication regimen complexity, particularly COPD‐specific complexity, and lifestyle factors such as smoking and alcohol use were significantly associated with nonadherence, highlighting the need for regimen simplification and targeted pharmacist‐led interventions.

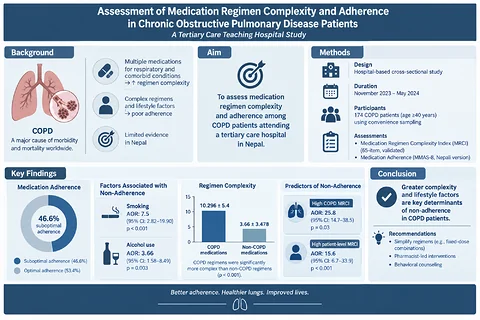

## 1 Introduction

Chronic obstructive pulmonary disease (COPD) is a progressive respiratory disorder characterized by airflow limitation resulting from abnormalities in the airways, bronchioles (chronic bronchitis), and alveoli (emphysema) [1]. Globally, COPD affects 65–80 million people, accounting for 10%–20% of the population. It is the third leading cause of death, responsible for 3.23 million deaths in 2019, and the seventh leading cause of poor health measured by disability‐adjusted life years [2]. In Southeast Asia, COPD‐associated mortality is projected to increase by 160% in the coming decades [3].

In Nepal, the prevalence of COPD is 11.7% [4]. The disease contributes to 43% of the national noncommunicable disease burden, 10% of noncommunicable diseases related deaths, and 3% of hospitalizations, with smoking and biomass fuel exposure identified as major risk factors [5]. Due to its chronic and heterogeneous nature, coupled with high prevalence, morbidity, and mortality, COPD represents a significant public health challenge, straining both healthcare system and providers [4, 5].

Advancing age, multiple comorbidities, and polypharmacy further increase regimen complexity in COPD patients. Most patients require multiple medications to manage both respiratory and non‐respiratory conditions [6]. The cornerstone of COPD management is the consistent use of prescribed therapies. Commonly prescribed medications include short‐acting beta‐agonists (SABA), long‐acting beta‐agonists (LABA), short‐acting muscarinic antagonists (SAMA), long‐acting muscarinic antagonists (LAMA), antibiotics, corticosteroids, mucolytics, and various fixed‐dose combinations [1]. These agents are available in multiple dosage forms, including oral, inhalation, and combination preparations that contribute to regimen complexity [7]. Such complexity often leads patients to deviate from prescribed regimens.

Medication regimen complexity is the quantification of the medication regimen, which can be obtained by using a 65‐item validated Medication Regimen Complexity Index (MRCI) tool based on dosage form (Section A), dosing frequency (Section B), and additional directions (Section C), where the sum of three sections gives the complexity index [8]. High regimen complexity is associated with suboptimal adherence, increased risk of medication errors, drug–drug interactions, adverse drug reactions, poor disease control, and treatment failure [6, 9, 10]. In Nepal, more than half of COPD patients are non‐adherent to their medications, often due to the regimen complexity and concerns about side effects [11]. Previous studies report that only one‐fourth of COPD patients demonstrate high medication adherence, with several personal and clinical factors contributing to poor compliance [12]. Nonadherence may take the form of incorrect dosing, missed doses, early discontinuation, or failure to fill prescriptions. As a result, non‐adherent patients are more likely to experience exacerbations compared with those who adhere to treatment [13].

Adherence in COPD is influenced by multiple factors. Patient‐related determinants include self‐esteem, psychological status, and comorbid conditions; drug‐related determinants include polypharmacy, regimen complexity, route of administration, and adverse effects; and healthcare‐related determinants include accessibility of medications and health services [14]. Evidence suggests that improved adherence can reduce mortality, minimize exacerbations, lower hospitalization rates, and decrease healthcare costs [15]. Despite these benefits, limited studies in Nepal have explored the association between regimen complexity and adherence in COPD, leaving an important evidence gap.

Studying medication regimen complexity and adherence can inform strategies to simplify regimens and improve treatment outcomes. The findings may guide healthcare providers, policymakers, and researchers in developing tailored interventions to reduce unnecessary complexity and improve adherence. Collaborative efforts among healthcare professionals can minimize inappropriate prescribing, whereas pharmacists play a key role in conducting medication reviews, simplifying regimens, and reducing hospital readmissions [16, 17]. Therefore, this study aimed to assess medication regimen complexity and adherence among COPD patients in Nepal.

## 2 Methods

### 2.1 Study Design, Setting, and Population

A hospital‐based cross‐sectional study was conducted at Manmohan Memorial Medical College and Teaching Hospital from November 2023 to May 2024. The hospital, a 300‐bed tertiary care facility located in the center of Kathmandu, Nepal, provides a wide range of specialty and some sub‐specialty services, ensuring evidence‐based healthcare delivery. Outpatient department (OPD) services were available from 9:00 a.m. to 4:00 p.m. The study population included patients of either gender, aged ≥40 years, with a physician‐confirmed diagnosis of COPD supported by post‐bronchodilator spirometry (FEV1/FVC < 0.70) in accordance with GOLD criteria [1] and a documented disease duration of at least 3 months, attending the OPD for follow‐up. The minimum disease‐duration criterion of three months was applied to ensure that patients had an established, stable pattern of COPD‐directed prescribing and sufficient exposure to their medication regimen for regimen complexity and adherence to be meaningfully assessed and to distinguish them from newly diagnosed patients whose treatment regimen may still be undergoing initial titration. Patients with asthma‐COPD overlap, newly diagnosed COPD, incomplete medical records, or those who declined to participate were excluded from the study.

### 2.2 Sample Size Calculation

The sample size was calculated by using the Cochrane formula, *n* = *z*^2 × *p* × *q*/*d*^2, where the prevalence of COPD, *p* = 11.7% [4], *q* = 1 − *p*, *d* = 0.05. Replacing the values in the formula, *n* = 1.96^2 × 0.117 × 0.883/0.05^2, *n* = 158. By adding a nonresponse rate of 10%, the total sample size becomes 174. A convenient sampling technique was used.

### 2.3 Data Collection

Data were collected through a combination of face‐to‐face interviews, review of OPD records, and prescription assessments. A semi‐structured questionnaire was developed by reviewing previously published studies in peer‐reviewed journals [8, 18, 19, 20] (Supporting Information). The questionnaire was administered during patient interviews to obtain sociodemographic and selected clinical information. Additional clinical characteristics were cross‐verified from OPD records. Furthermore, a structured pro forma was used to document and analyze the medications prescribed for COPD management.

### 2.4 Outcome Measures

Medication regimen complexity was assessed using a validated 65‐item MRCI tool [8], originally developed for patients with COPD. The tool comprises three sections: Section A (dosage form), Section B (dosing frequency), and Section C (additional directions), which together generates the total MRCI score. A higher MRCI score indicates greater regimen complexity. Both COPD‐related medications and non‐COPD medications prescribed for comorbid conditions were reviewed, and the MRCI was calculated using the University of Colorado's electronic MRCI data capture tool [21].

The Morisky Medication Adherence Scale (MMAS‐8) was employed to assess patients’ medication adherence. This is a widely validated and reliable tool with strong predictive validity for measuring adherence in patients with chronic diseases [20]. The instrument comprises eight items: the first seven with dichotomous (Yes/No) responses and the eighth with a five‐point Likert scale. On the basis of the scoring system, a total score of <6 indicates low adherence, 6 and 7 indicate moderate adherence, and a score of 8 indicates high adherence. To adapt the scale for use in Nepal, a cross‐language equivalence procedure was followed. The MMAS‐8 was initially translated into Nepali and then back‐translated into English by two independent pharmacists not involved in the study. The Nepali version demonstrated good internal consistency (Cronbach's alpha = 0.84) in a pilot study involving 16 participants.

### 2.5 Explanatory Variables

Although the eligibility criterion for enrollment was age ≥40 years, no enrolled patient was aged between 40 and 44 years; the youngest participant recruited was 45 years old. For descriptive and bivariate analyses, age was therefore categorized on the basis of the observed distribution as 45 to ≤65 years and >65 years. Explanatory variables considered in this study were age (categorized as 45 to ≤65 and >65), gender (male, female), occupation (professional work, agriculture, homemakers, business, unemployed), and education status (illiterate, primary education, secondary education, or above), smoking status (current smoker, former smoker, nonsmoker), alcohol use (current alcoholic, former alcoholic, non‐alcoholic), and comorbidities (presence/absence).

#### 2.5.1 Statistical Analysis

The data collected were carefully reviewed for accuracy and completeness. Data entries were performed using EpiData software version 3.1, and all data were subsequently exported to SPSS version 22 for statistical analysis. Descriptive statistics, including frequency, percentage, mean, and standard deviation (SD), were used to summarize the data. The *χ* ^2^ test was performed at a 5% level of significance to identify the association of comorbid conditions with non‐COPD MRCI, as well as the factors associated with medication adherence. Factors that showed statistical significance (*p* < 0.05) in the *χ* ^2^ test were included in the final model of multivariable logistic regression to calculate adjusted odds ratios (aOR). Prior to multivariable analysis, multicollinearity among the selected independent variables was assessed using the variance inflation factor (VIF), with a VIF greater than five considered indicative of multicollinearity.

### 2.6 Operational Definitions

**Medication regimen complexity**: Quantification of a patient's prescribed medications based on three components: dosage form (Section A), dosing frequency (Section B), and additional directions (Section C).

**COPD‐specific MRCI**: Medication regimen complexity of medications prescribed specifically for COPD. Scores are categorized as low (≤10) or high (>10).

**Non‐COPD MRCI**: Medication regimen complexity of medications prescribed for comorbid conditions and other over‐the‐counter (OTC) drugs. Scores are categorized as low (≤4) or high (>4).

**Patient level MRCI**: Overall medication regimen complexity, including all prescribed and OTC medications. Scores are categorized as low (≤14) or high (>14).

**Level of medication adherence**: Defined as the extent to which a patient's behavior aligns with healthcare provider recommendations. Categorized as suboptimal (<6) and optimal (6–8) based on the MMAS‐8 scale.

**Smoking status**: Classified per the World Health Organization STEPwise approach to surveillance (STEPS) [22] as current smoker (smoking at the time of interview, either daily or occasionally), former smoker (had smoked ≥100 cigarettes/equivalent tobacco products in their lifetime but not smoked at the time of interview), or nonsmoker (never smoked).

**Alcohol use**: Classified analogously to smoking status [22] as current alcohol user (consuming alcohol at the time of interview, either daily or occasionally), former alcohol user (had consumed alcohol regularly in the past but had abstained for at least the preceding 12 months), or nonuser of alcohol (never consumed alcohol).

### 2.7 Ethical Considerations

Ethical approval was obtained from the Institutional Review Committee of the Central Institute of Science and Technology (IRC CiST‐Ref No: 29/2080/81), and the study was conducted in accordance with the Helsinki Ethical Guidelines. Permission for data collection was granted by Manmohan Memorial Medical College and Teaching Hospital (Ref No: 437/080/081). Authorization and a license to use the Morisky Medication Adherence Scale MMAS‐8 (Certificate Number: 1407‐7433‐3185‐4820‐5609) were obtained for this study. Written informed consent was obtained from all participants, and thumbprints were collected from illiterate participants. Participation was entirely voluntary, and sufficient time was provided for participants to read and respond to the questions.

## 3 Results

Table 1 shows the baseline characteristics of COPD patients and their distribution across medication adherence categories. The mean age of participants was (Mean ± SD 68.41 + 10.756) years, with a predominance of elderly patients (>65 years; 60.9%). However, adherence did not differ significantly by age (*p* = 0.606). More than half of the patients were female (53.4%), but gender showed no significant association with adherence (*p* = 0.485). Notably, 27.6% of patients were unemployed, 50% were illiterate, and both factors did not demonstrate significant association with adherence (*p* = 0.409 and *p* = 0.114, respectively).

**TABLE 1:** Baseline characteristics and bivariate analyses by medication adherence (*n* = 174).

		Medication adherence		
	Total *n* = 174 (100%)	Suboptimal (*n* = 81, 46.6%)	Optimal (*n* = 93, 53.4%)	
Variables	*n* (%)	*n* (%)	*n* (%)	*p* value
**Age (Years)**				
45 to ≥65	68 (39.1)	30 (44.1)	38 (55.9)	0.606
>65	106 (60.9)	51 (48.1)	55 (51.9)	
**Gender**				
Male	81 (46.6)	40 (49.4)	41 (50.6)	0.485
Female	93 (53.4)	41 (44.1)	52 (55.9)	
**Occupation**				
Professional work	28 (16.1)	10 (35.7)	18 (64.3)	0.114
Agriculture	41 (23.6)	20 (48.8)	21 (51.2)	
Homemakers	45 (25.9)	21 (46.7)	24 (53.3)	
Business	12 (6.9)	2 (16.7)	10 (83.3)	
Unemployed	48 (27.6)	28 (58.3)	20 (41.7)	
**Education status**				
Illiterate	87 (50.0)	44 (50.6)	43 (49.4)	0.409
Primary education	77 (44.3)	32 (41.6)	45 (58.4)	
Secondary education or above	10 (5.2)	6 (60.0)	4 (40.0)	
**Smoking status**				
Current smoker	35 (20.1)	24 (68.6)	11 (31.4)	<0.001*
Former smoker	84 (48.3)	44 (52.4)	40 (47.6)	
Nonsmoker	55 (31.6)	13 (23.6)	42 (76.4)	
**Alcohol use**				
Current use of alcohol	35 (20.1)	23 (65.7)	12 (34.3)	0.003*
Non use of alcohol	89 (51.1)	31 (34.8)	58 (65.2)	
Former alcohol user	50 (28.7)	27 (54)	23 (46)	
**Presence of comorbidity**				
Present	122 (70.1)	58 (47.5)	64 (52.5)	0.689
Absent	52 (29.9)	23 (44.2)	29 (55.8)	

*p value < 0.05.

Lifestyle‐related factors were strongly associated with adherence. Current smokers (20.1%) exhibited the highest rate of suboptimal adherence (68.6%), compared to only 23.6% among nonsmokers (*p* < 0.001). Similarly, current alcohol users (20.1%) had higher rates of nonadherence (65.7%) compared to nonusers of alcohol (34.3%; *p* = 0.003). Comorbidities were highly prevalent, with 70.1% of the patients reporting at least one chronic condition in addition to COPD but did not significantly affect the adherence (*p* = 0.689).

Table 2 shows that combination therapy was the predominant approach among COPD patients, with 59.2% receiving triple therapy comprising LABA + ICS + LAMA. Likewise, 15.5% were prescribed with LABA + ICS and 10.9% with LAMA + LAMA. In contrast, monotherapy was limited to 9.2% receiving SABA alone and 5.2% receiving LAMA alone.

**TABLE 2:** Monotherapy and combination therapy of drugs (*n* = 174).

Drug category	*n* (%)
Combination therapy	
LABA + ICS + LAMA	103 (59.2)
LABA + ICS	27 (15.5)
LABA + LAMA	19 (10.9)
Monotherapy	
SABA only	16 (9.2)
LAMA only	9 (5.2)

Abbreviations: ICS: inhaled corticosteroids; LABA: long‐acting beta‐agonists; LAMA: long‐acting muscarinic antagonists; SABA: short‐acting beta‐agonists.

As shown in Table 3, the mean number of total medications was 4.81 ± 2.61 per patient. Nearly one‐third of patients (31.2%) were prescribed more than six medications overall. Although two medications were most commonly prescribed for COPD (32.2%), a considerable proportion (16.1%) required more than six. For non‐COPD conditions, most patients received one (20.7%) to two (24.1%) medications, though 5.8% had more than six.

**TABLE 3:** Number of medications (*n* = 174).

Number of medications(s)	COPD *n* (%)	Non‐COPD *n* (%)	Total *n* (%)
0	—	47 (27.0)	—
1	30 (17.2)	36 (20.7)	12 (6.9)
2	56 (32.2)	42 (24.1)	15 (8.6)
3	32 (18.4)	22 (12.6)	31 (17.8)
4	28 (16.7)	14 (8.0)	33 (19.0)
5	19 (10.9)	3 (1.7)	29 (16.7)
>6	28 (16.1)	10 (5.8)	49 (31.2)
Mean ± SD	2.87 ± 1.453	2.87 ± 1.453	4.81 ± 2.61

Abbreviations: COPD, chronic obstructive pulmonary disease; SD, standard deviation.

Table 4 shows the distribution of medication regimen complexity among patients. More than half of the patients (56.9%) had low complexity for COPD‐specific medications, whereas 43.1% had high complexity. For non‐COPD medications, 66.7% fell into the low complexity category, compared to 33.3% with high complexity. At the overall patient level, 58.0% exhibited low regimen complexity, whereas 42.0% demonstrated high complexity.

**TABLE 4:** Medication regimen complexity (*n* = 174).

Variables	Low *n* (%)	High *n* (%)
COPD MRCI	99 (56.9)	75 (43.1)
Non‐COPD MRCI	116 (66.7)	58 (33.3)
Patient level MRCI	101 (58.0)	73 (42.0)

Abbreviations: COPD, chronic obstructive pulmonary disease; MRCI, Medication Regimen Complexity Index.

Table 5 shows the mean ± SD values for the MRCI scores and sub‐scores related to COPD medications, non‐COPD medications, and the overall patient‐level MRCI. The mean MRCI score was 10.296 ± 5.4 for COPD medications and 3.66 ± 3.478 for non‐COPD medications, resulting in a combined patient‐level MRCI of 13.9 ± 6.64.

**TABLE 5:** Mean ± standard deviation (SD) of Medication Regimen Complexity Index (MRCI) score and sub‐scores.

Variables	COPD MRCI	non‐COPD MRCI	Patient level MRCI
MRCI score	10.296 ± 5.4	3.66 ± 3.478	13.9 ± 6.64
Dosage form MRCI	2.87 ± 1.453	0.85 ± 0.745	—
Dosing frequency MRCI	4.89 ± 2.549	2.316 ± 2.488	—
Additional directions from MRCI	4.49 ± 2.7	0.51 ± 0.810	—
Medicine count	2.87 ± 1.453	1.93 ± 1.99	4.81 ± 2.61

Abbreviation: COPD, chronic obstructive pulmonary disease.

For COPD medications, sub‐scores included dosage form (2.87 ± 1.453), dosing frequency (4.89 ± 2.549), and additional directions (4.49 ± 2.7). In contrast, non‐COPD medications had lower sub‐scores for dosage form (0.85 ± 0.745), dosing frequency (2.316 ± 2.488), and additional directions (0.51 ± 0.810). The average number of medicines prescribed was 2.87 ± 1.453 for COPD, 1.93 ± 1.99 for non‐COPD, and 4.81 ± 2.61 at the overall patient level.

Table 6 shows association between various comorbidities and both the non‐COPD MRCI and the total medicine count. Dyslipidemia, coronary artery disease (CAD), and benign prostatic hyperplasia (BPH) showed strong associations with higher non‐COPD MRCI scores (*p* < 0.001; *p* = 0.045; and *p* = 0.026, respectively) and elevated medicine counts (*p* < 0.001; *p* = 0.02; and *p* = 0.001, respectively). Hypertension was also associated with higher MRCI (*p* = 0.031), though not with medicine count. Cor pulmonale showed a significant increase in medicine count (*p* = 0.015) without affecting MRCI. Other conditions such as anemia, GERD, and thyroid disorders did not significantly impact either parameter.

**TABLE 6:** Association of comorbid conditions with non‐chronic obstructive pulmonary disease (COPD) Medication Regimen Complexity Index (MRCI) and total medicine count.

Comorbid conditions	Non‐COPD MRCI *n* (%)			Medicine count	
	Low	High	*p* value	Mean ± SD	*p* value
Hypertension	48 (62.3)	29 (37.7)	0.031*	2.68 ± 1.956	0.836
Diabetes	15 (53.6)	13 (46.4)	0.870	2.61 ± 1.571	0.900
Dyslipidemia	3 (14.3)	18 (85.7)	<0.001*	4.24 ± 2.528	<0.001**
Anemia	4 (57.1)	3 (42.9)	1.00	2.57 ± 1.272	0.915
BPH	6 (31.6)	13 (68.4)	0.026*	4 ± 2.728	0.001*
GERD	5 (45.5)	6 (54.5)	0.541	2.73 ± 1.348	0.887
Thyroid disorder	5 (45.5)	6 (54.5)	0.541	3.27 ± 2.611	0.687
Coronary artery disease	1 (14.3)	6 (85.7)	0.045*	4.86 ± 1.773	0.002*
Cor pulmonale	1 (16.7)	5 (85.3)	0.90	4.50 ± 2.345	0.015*

*Note:* Chi‐square and independent *t*‐test (*p < 0.05, **p < 0.01).

Abbreviations: BPH: benign prostate hyperplasia; GERD; gastroesophageal reflux disease; SD, standard deviation.

Table 7 shows the unadjusted aOR for factors associated with medication nonadherence among COPD patients. After adjusting for potential confounders, current smokers (aOR: 7.5; 95% CI: 2.82–19.90; *p* < 0.001) and former smokers (aOR: 3.76; 95% CI: 1.717–8.271; *p* = 0.001) had significantly higher odds of nonadherence compared to nonsmokers.

**TABLE 7:** Unadjusted and adjusted odds ratios (ORs) for factors associated with medication nonadherence.

Characteristics	*p* value	Unadjusted OR	95% CI	*p* value	Adjusted OR	95% CI
**Smoking habit**						
Nonsmoker	**Ref**.			**Ref**.		
Current smoker	<0.001**	7.049	(2.7–18.16)	<0.001**	7.5	(2.82–19.90)
Former smoker	0.001*	3.55	(1.67–7.5)	0.001*	3.76	(1.717–8.271)
**Alcohol drinking habit**						
Nonuse of Alcohol	**Ref**.			**Ref**.		
Current alcohol user	0.02*	3.588	(1.57–8.16)	0.003*	3.66	(1.578–8.49)
Former alcohol user	0.029*	2.16	(1.08–4.4)	0.032*	2.25	(1.07–4.748)
**COPD MRCI**						
Low		**Ref**.		**Ref**.		
High	<0.001**	31.0	(18.4–51.4)	0.03*	25.8	(14.7–38.5)
**Non‐COPD MRCI**						
Low		**Ref**.		**Ref**.		
High	0.006*	0.39	(0.20–0.74)	0.04	0.32	(0.17–0.64)
**Patient‐level MRCI**						
Low		**Ref**.		**Ref**.		
High	<0.001**	17.6	(8.2–38.0)	<0.001**	15.6	(6.7–33.9)

*Note:* Ref = reference group.

Abbreviations: CI, confidence interval; COPD, chronic obstructive pulmonary disease; MRCI, Medication Regimen Complexity Index; OR, odds ratio.

*p value< 0.05.

**p value <0.01.

Similarly, current alcohol users (aOR: 3.66; 95% CI: 1.578–8.49; *p* = 0.003) and former alcohol users (aOR: 2.25; 95% CI: 1.07–4.748; *p* = 0.032) were also more likely to be non‐adherent than patients who did not use alcohol.

A higher COPD MRCI was a strong predictor of nonadherence (aOR: 25.8; 95% CI: 14.7–38.5; *p* = 0.03), as was a high overall patient‐level MRCI (aOR: 15.6; 95% CI: 6.7–33.9; *p* < 0.001). In contrast, a higher non‐COPD MRCI was associated with significantly lower odds of nonadherence (aOR: 0.32; 95% CI: 0.17–0.64; *p* = 0.04), suggesting that patients managing complex non‐COPD regimens may demonstrate better adherence.

## 4 Discussion

This study assessed the complexity of medication regimens and their association with adherence among COPD patients attending a tertiary care hospital in Nepal. The findings revealed that COPD‐specific regimens had higher complexity than non‐COPD medications, and increasing regimen complexity was strongly associated with adherence. Moreover, lifestyle factors such as smoking and alcohol consumption were significant predictors of nonadherence.

The findings indicate that COPD regimens are inherently more complex compared to therapies for comorbid conditions, possibly due to the use of multiple inhalers, different dosage forms, and varied dosing schedules. This observation is consistent with studies showing that the MRCI is a useful predictor of treatment burden in COPD [6, 7]. In particular, Negewo et al. [6] and Prosser and Bollmeier [7] similarly reported that inhaled COPD‐directed therapy consistently contributed a larger share of total regimen complexity than medications prescribed for comorbid conditions, largely driven by the dosage‐form and additional‐directions sub‐scores associated with multiple inhaler devices, spacers, and device‐specific administration techniques, rather than by the number of medications alone. This mirrors the present findings, in which the dosage‐form and additional‐directions sub‐scores for COPD medications were each severalfold higher than the corresponding non‐COPD sub‐scores. Previous studies have demonstrated that higher regimen complexity contributes to poor disease control, medication error, and increased risk of exacerbations and hospitalizations [10, 17]. The strong association between high COPD‐specific and patient‐level MRCI and nonadherence observed in this study reinforces the clinical relevance of this complexity, because patients confronted with multiple inhaler devices and administration techniques for COPD alone may struggle to sustain correct and consistent use, independent of the burden imposed by comorbid‐condition medications.

This study further confirmed the inverse relationship between regimen complexity and adherence. Similar associations have been reported in both high‐ and low‐income settings, where patients with complex regimens were less likely to follow treatment recommendations [9, 23]. A prospective study from China also demonstrated that increased medication regimen complexity was independently associated with poorer adherence and worse clinical outcomes, including more frequent exacerbations and hospitalizations, in COPD patients [24].

Lifestyle factors also emerged as a strong predictor of adherence. Current smokers and alcohol consumers were more likely to be non‐adherent, consistent with prior Nepalese and international studies [11, 12, 14]. A comparable secondary‐care cohort from Northern Ireland similarly identified current smoking status, alongside perceived medication ineffectiveness and depressed mood, as an independent correlate of poor adherence in COPD [25], suggesting that the association observed in the present study reflects a broader, previously documented pattern rather than a setting‐specific finding. This behavior may contribute to poor disease insight, reduced motivation to adhere, or competing priorities that undermine treatment adherence; ongoing tobacco and alcohol use may also reflect lower health literacy or weaker engagement with the healthcare system, both of which independently predispose to medication nonadherence. Addressing these behavioral determinants is therefore crucial, and comprehensive COPD management should integrate behavioral counseling, smoking cessation, and alcohol reduction programs alongside pharmacological therapy.

Multimorbidity was highly prevalent among COPD patients in this study, aligning with the global literature that highlights the close association between COPD and cardiovascular, metabolic, and other chronic conditions [26, 27]. Where comorbidities generally contribute to greater regimen complexity, some studies have observed that patients with multiple conditions may be more adherent, probably due to frequent healthcare interactions and increased disease awareness [16].

Among the comorbidities examined, dyslipidemia and benign prostatic hyperplasia (BPH) showed the strongest associations with higher non‐COPD MRCI and medicine count, and these findings carry specific clinical relevance. COPD and dyslipidemia frequently coexist, likely reflecting the shared contribution of chronic systemic inflammation and common risk factors such as smoking and physical inactivity to both airway and metabolic‐cardiovascular derangement; a recent comprehensive comorbidity study similarly reported a significantly higher prevalence of dyslipidemia among COPD patients compared with non‐COPD controls [28]. Management of dyslipidemia typically adds one or more lipid‐lowering agents with distinct dosing schedules and monitoring requirements, which directly increases both dosing frequency and additional‐directions sub‐scores of the non‐COPD MRCI. The association with BPH is clinically noteworthy because several COPD medications used in this cohort, particularly SAMA/LAMA, have anticholinergic activity: a large population‐based study demonstrated that inhaled anticholinergic therapy increases the risk of acute urinary retention in older men with COPD, with the risk further amplified in those with preexisting BPH [29]. In such patients, BPH is frequently managed with alpha‐blockers or 5‐alpha‐reductase inhibitors, adding further medications and monitoring instructions on top of an already anticholinergic‐heavy COPD regimen. Clinicians should therefore be alert to this drug–disease interaction, and pharmacist‐led medication reviews may be particularly valuable for older male COPD patients with coexisting BPH to balance respiratory symptom control against urological risk.

A major strength of this study lies in the use of validated tools to comprehensively assess both regimen complexity and adherence in a Nepalese population, thereby contributing valuable local evidence in an area where data are scarce. Nevertheless, several limitations should be acknowledged. The relatively small sample size of 174 patients, calculated primarily to estimate the prevalence of medication nonadherence, limited the precision of some subgroup and comorbidity‐specific estimates, as reflected in the wide confidence intervals for several aOR; this should be considered when generalizing the magnitude of these associations, and larger multicenter studies are needed to confirm them. The cross‐sectional design precludes causal inference between regimen complexity and adherence. The reliance on self‐reported adherence may be a subject to recall and social desirability bias. Likewise, the use of convenience sampling in a single center may limit the generalizability of the findings to broader healthcare settings in Nepal. Finally, important unmeasured factors such as health literacy and psychological factors of adherence were not comprehensively evaluated and may have influenced the outcomes. Future studies incorporating a validated socioeconomic classification are recommended.

## 5 Conclusion

This study demonstrates that COPD‐specific regimens were comparatively more complex, and higher regimen complexity was significantly associated with poorer adherence. Lifestyle factors, particularly smoking and alcohol consumption, also contributed substantially to nonadherence. These findings highlight the urgent need to simplify COPD pharmacotherapy through fixed‐dose combinations, long‐acting inhalers, and rational prescribing. Integrating pharmacist‐led interventions, inhaler technique training, and behavioral counseling into routine care may further enhance adherence and optimize treatment outcomes. Future longitudinal and interventional studies are warranted to confirm causal relationships and evaluate the effectiveness of these adherence‐support strategies.

## Author Contributions

**Kabita Bhandari**: conceptualization, methodology, data curation, investigation, visualization, project administration, resources, writing – original draft. **Nirmal Raj Marasine**: conceptualization, methodology, data curation, validation, formal analysis, supervision, visualization, project administration, resources, writing – original draft, writing – review and editing. **Chakra Dev Bist**: writing – review and editing. **Poonam Pant**: supervision.

## Ethics Statement

Ethical approval was obtained from the Institutional Review Committee of the Central Institute of Science and Technology (Ref no: 29/2080/81), and the study followed the Helsinki Ethical Guidelines. Participation was voluntary, and sufficient time was provided for participants to read and answer the questions.

## Consent

Written informed consent was obtained from all patients, and thumbprints were collected from illiterate patients.

## Conflicts of Interest

The authors declare no conflicts of interest.

## Supporting information




**Supporting file 01:** puh270449‐sup‐0001‐S1_2.docx

## Data Availability

The datasets used and/or analyzed during the current study are available from the corresponding author on reasonable request.
