# Supplementary material for: Assessment of Medication Regimen Complexity and Adherence in Chronic Obstructive Pulmonary Disease Patients at a Tertiary Care Teaching Hospital
Source: Public Health Chall. 2026 Nov 7;5(4):e70449. doi: 10.1002/puh2.70449 (PMC13744624; doi:10.1002/puh2.70449)
Supplement: Supplementary file 1 [file PUH2-5-e70449-s001.docx]

**Data collection form**

**Section I: Socio-demographic characteristics**

| S.N | Questions | Response |
| --- | --- | --- |
| 1. | Age |  |
| 2. | Gender | Male Female |
| 3. | Occupation | Government job Non-government Job    Agriculture Homemakers    Business Unemployed |
| 4. | Education | Illiterate  Primary education    Secondary education  Graduate  Postgraduate |
| 5. | Ethnicity | Brahmin Chettri    Janajati Dalit    Others |
| 6. | Religion | Hinduism Buddhism  Christianity Islam    Others |
| 7. | Marital status | Unmarried Married    Divorced Widow/widower |

Participant code: Address of participant:

**Section II: Lifestyle factors**

| Questions | Response |
| --- | --- |
| 1. Do you smoke? | Current smoker Former smoker    Non-smoker |
| 1.1 If you are a current smoker, how many cigarettes do you smoke per day? | ……………………. |
| 2. Do you take alcohol? | Yes, N No |

**Section III: Clinical factors**

| Questions | Response |
| --- | --- |
| 1. Comorbidity | Yes No |
| 1.1 If yes, mention here | …………………….. |

**ANNEX III: Drugs Prescribed for COPD**

S. N

Drugs used

| Brand Name | Generic name and dose | Dosage form | Route | Frequency | Indication | Therapeutic Class |
| --- | --- | --- | --- | --- | --- | --- |
|  |  |  |  |  |  |  |
|  |  |  |  |  |  |  |
|  |  |  |  |  |  |  |
|  |  |  |  |  |  |  |
|  |  |  |  |  |  |  |
|  |  |  |  |  |  |  |
|  |  |  |  |  |  |  |

Number of drugs taken:

**ANNEX IV: Medication regimen complexity index**

Circle the weighting corresponding to each dosage form (ONCE ONLY) present in the regimen.

| Dosage form | | Weighting |
| --- | --- | --- |
| ORAL | Capsules/tablets | 1 |
|  | Gargles/ Mouthwashes | 2 |
|  | Gum/lozenges | 2 |
|  | Liquids | 2 |
|  | Powders/granules | 2 |
|  | Sublingual sprays /Tabs | 2 |
| TOPICAL | Creams/gels/ointments | 2 |
|  | Dressings | 3 |
|  | Paints/solution | 2 |
|  | Pastes | 3 |
|  | Patches | 2 |
|  | Sprays | 1 |
| EAR, EYE, NOSE | Ear drops/creams/ointments | 3 |
|  | Eye drops | 3 |
|  | Eye gels/ointments | 3 |
|  | Nasal Drops/ creams/ ointment | 3 |
|  | Nasal sprays | 2 |
| INHALATION | Accuhalers | 3 |
|  | Aerolizers | 3 |
|  | Metered dose inhalers | 4 |
|  | Nebulizer | 5 |
|  | Oxygen concentrator | 3 |
|  | Turbuhalers | 3 |
|  | Other dry powder inhalers | 3 |
| OTHERS | Dialysate | 5 |
|  | Enemas | 2 |
|  | Injection: Prefilled ampules/vials | ¾ |
|  | Pessaries | 3 |
|  | Patient controlled analgesia | 2 |
|  | Suppositories | 2 |
|  | Vaginal cream | 2 |
| TOTAL FOR SECTION A | | |

For each medication in the regimen tick a box ( ) corresponding to the dosing frequency. Then add the number of ticks in each category and multiply by the assigned weighting. In cases where there is no exact option choose the best option.

| Dosing frequency | Medications | | | | | | | | Total | Weighting | Weighting * No. of medications |
| --- | --- | --- | --- | --- | --- | --- | --- | --- | --- | --- | --- |
| Once daily |  |  |  |  |  |  |  |  |  | 1 |  |
| Once daily prn |  |  |  |  |  |  |  |  |  | 0.5 |  |
| Twice daily |  |  |  |  |  |  |  |  |  | 2 |  |
| Twice daily prn |  |  |  |  |  |  |  |  |  | 1 |  |
| Three times daily |  |  |  |  |  |  |  |  |  | 3 |  |
| Three times daily prn |  |  |  |  |  |  |  |  |  | 1.5 |  |
| Four times daily |  |  |  |  |  |  |  |  |  | 4 |  |
| Four times daily prn |  |  |  |  |  |  |  |  |  | 2 |  |
| q 12 hr |  |  |  |  |  |  |  |  |  | 2.5 |  |
| q 12hr prn |  |  |  |  |  |  |  |  |  | 1.5 |  |
| q 8 hr |  |  |  |  |  |  |  |  |  | 3.5 |  |
| q 8 hr prn |  |  |  |  |  |  |  |  |  | 2 |  |
| q 6 hr |  |  |  |  |  |  |  |  |  | 4.5 |  |
| q 6 hr prn |  |  |  |  |  |  |  |  |  | 2.5 |  |
| q 4 hr |  |  |  |  |  |  |  |  |  | 6.5 |  |
| q 4 hr prn |  |  |  |  |  |  |  |  |  | 3.5 |  |
| q 2 hr |  |  |  |  |  |  |  |  |  | 12.5 |  |
| q 2 hr prn |  |  |  |  |  |  |  |  |  | 6.5 |  |
| prn/sos |  |  |  |  |  |  |  |  |  | 0.5 |  |
| On alternate day or less frequently |  |  |  |  |  |  |  |  |  | 2 |  |
| Oxygen prn |  |  |  |  |  |  |  |  |  | 1 |  |
| Oxygen < 15 hrs |  |  |  |  |  |  |  |  |  | 2 |  |
| Oxygen > 15 hrs |  |  |  |  |  |  |  |  |  | 3 |  |
| TOTAL FOR SECTION B | | | | | | | | | | | |

Tick a box () corresponding to the additional directions, if present in the regimen. Then add the number of ticks in each category and multiply by the assigned weighting.

| Additional directions | Medications | | | | | | | | | Total | | Weighting | | Weighting * NO. of medications | |
| --- | --- | --- | --- | --- | --- | --- | --- | --- | --- | --- | --- | --- | --- | --- | --- |
| Break /crush the tablet |  |  |  |  |  |  |  |  |  | | 1 | |  | |  |
| Dissolve tablet or powder |  |  |  |  |  |  |  |  |  | | 1 | |  | |  |
| Multiple units at one time (2puffs, 2 tabs) |  |  |  |  |  |  |  |  |  | | 1 | |  | |  |
| Variable dose (eg: 1-2 caps,2-3 puffs) |  |  |  |  |  |  |  |  |  | | 1 | |  | |  |
| Take or use at specified times (eg. Mane nocte, 8 am) |  |  |  |  |  |  |  |  |  | | 1 | |  | |  |
| Relation to food (eg. Pc, ac, with food) |  |  |  |  |  |  |  |  |  | | 1 | |  | |  |
| Take with specific fluid |  |  |  |  |  |  |  |  |  | | 1 | |  | |  |
| Take / use as directed |  |  |  |  |  |  |  |  |  | | 2 | |  | |  |
| Tapering/increasing dose |  |  |  |  |  |  |  |  |  | | 2 | |  | |  |
| Alternating Dose (eg .one mane and two nocte, one/two on alternate days) |  |  |  |  |  |  |  |  |  | | 2 | |  | |  |
| TOTAL FOR SECTION C | | | | | | | | | | | | | | | |

**ANNEX V: Morisky medication adherence scale 8 items (MMAS-8)**

| S.N | Questions | Yes =0 | NO=1 |
| --- | --- | --- | --- |
| 1. | Do you sometimes forget to take your [health concern] medication(s)? |  |  |
| 2. | People sometimes miss taking their medications for reasons other than forgetting. Thinking over the past two weeks, were there any days when you did not take your [health concern] medication(s)? |  |  |
| 3. | Have you ever cut back or stopped taking your medication(s) without telling your doctor, because you felt worse when you took it? |  |  |
| 4. | When you travel or leave home, do you sometimes forget to bring along your [health concern] medication(s)? |  |  |
| 5. | Did you take your [health concern] medication(s) yesterday? (or the last time you were supposed to take it?) |  |  |
| 6. | When you feel like your [health concern] is under control, do you sometimes stop taking your medication(s)? |  |  |
| 7. | Taking medication(s) every day is a real inconvenience for some people. Do you ever feel hassled about sticking to your [health concern] treatment plan? |  |  |
| 8. | How often do you have difficulty remembering to take all your medication(s)?  (Please circle your answer below)  Never/Rarely ....................................... a  Once in a while..................................... b  Sometimes..............................................c Usually.................................................. d  All the time.............................................e |  |  |
